# Bridging the Micro-to-Nano Gap: A Thermo-Coupled Broadband Photodetector for Photon–Phonon Dual Harvesting

**DOI:** 10.3390/s26216696

**Published:** 2026-10-23

**Authors:** Halil Ibrahim Yavuz, Arda Tuna Vit, Buse Uzunçarşılı, Gün Güven, Mehmet Ege Balkan

**Affiliations:** 1 Materials Science and Nanotechnology Engineering, Faculty of Engineering and Applied Science, Yeditepe University, Istanbul 34755, Türkiye; 2 Nano7ech Laboratory, Faculty of Engineering and Applied Science, Yeditepe University, Istanbul 34755, Türkiye; 3 Department of Physics, Faculty of Engineering and Applied Science, Yeditepe University, Istanbul 34755, Türkiye

**Keywords:** self-powered photodetector, upconversion nanoparticles, photon–phonon dual-harvesting, thermoelectric modulation, defect engineering, Type-II heterojunction, hybrid integration

## Abstract

**Highlights:**

**What are the main findings?**

- A monolithic UPC/TiO_2_/Bi_2_Te_3_ heterostructure successfully captures parasitic upconversion waste heat and converts it into a 304 µV dynamic thermoelectric potential, effectively modulating the built-in junction barrier.
- Despite incorporating severe realistic interface defect penalties and lattice mismatches, this thermo-coupled dual-harvesting mechanism sustains a highly stable Fill Factor of 85.4% under self-powered, zero-bias operation.

**What are the implications of the main findings?**

- By utilizing a deliberate Bi_2_Te_3_ thermal bottleneck, the architecture transforms waste heat from a parasitic vulnerability into an operational driving force, successfully circumventing the efficiency saturation limits of traditional detectors.
- The newly proposed hybrid thick-film and thin-film (polymer matrix) integration strategy provides a highly practical blueprint for overcoming mechanical delamination and cracking across extreme micro-to-nano dimensional gaps.

**Abstract:**

Self-powered broadband photodetectors face severe thermal degradation limits at high excitation powers due to the parasitic heat generated by upconversion nanoparticles (UPC). To overcome this thermal bottleneck, we propose a monolithic UPC/FTO/TiO_2_/Bi_2_Te_3_ heterostructure that simultaneously harvests both photonic and thermal energies at the integrated multilayer scale. Unlike conventional architectures that suffer from efficiency saturation, our design utilizes the narrow-bandgap Bi_2_Te_3_ layer as a deliberate thermal confinement bottleneck. This configuration converts the localized multi-phonon waste heat into a dynamic thermoelectric potential (V_TE_ ≈ 304 µV) via the Seebeck effect, which synergistically modulates the built-in potential of the Type-II staggered junction. To ensure high physical fidelity, our model explicitly incorporates non-ideal interface physics, quantifying the voltage drops induced by lattice mismatch, deep-level trap states, and Shockley–Read–Hall (SRH) recombination centers at the heterojunction. Reduced-order multiphysics drift-diffusion and heat transfer boundary value problem (BVP) simulations demonstrate that this thermo-coupled mechanism provides a robust stabilizing effect against thermal degradation. Within our strictly bounded non-ideal model, the device achieves a realistic short-circuit current density of 11.45 mA/cm^2^, an open-circuit voltage of 0.342 V, and a Fill Factor of 85.4% under zero-bias operation. Furthermore, to bridge the massive lithographic gap between the bulk-like UPC layer (114.5 µm) and the thin-film TiO_2_ (4 µm), we propose a hybrid thick-film integration strategy to prevent thermo-mechanical delamination and structural cracking. Our findings provide a new design paradigm for achieving highly stable detectivity in self-powered optoelectronic devices by transforming thermal losses from a parasitic vulnerability into an operational compensating force.

## 1. Introduction

### 1.1. Demand for Self-Powered Autonomous Sensors in IoT Ecosystems

The rapid global proliferation of the Internet of Things (IoT), wearable electronic hardware, smart home automation, and autonomous environmental monitoring networks has unprecedentedly amplified the demand for seamless, independently operating, next-generation sensing technologies [1,2,3,4,5,6]. Traditional sensor architectures heavily rely on external batteries or an external bias voltage to sustain their operational continuity [7]. However, integrating these external power units into sensor nodes dramatically increases the total weight and volume (form factor) of the devices, thereby compromising the ergonomics, particularly of wearable and flexible hardware [8].

Furthermore, in large-scale networks comprising thousands of nodes, battery procurement, regular replacement, and maintenance labor create massive operational expenditures (economic burdens) that threaten the sustainability of the system [9]. In addition to these weight and cost disadvantages, the limited lifecycle of batteries, periodic maintenance requirements, difficulties of replacement under harsh field conditions, and environmental toxicity risks during disposal processes severely constrain the autonomous character of these systems [7,9]. To overcome this critical bottleneck, developing self-powered sensor topologies capable of directly converting microscopic ambient energy sources (such as light, heat, or mechanical vibrations) into a functional electrical signal has emerged as one of the most vital research frontiers in modern optoelectronic engineering [10,11].

### 1.2. Spectral Blindness of Wide-Bandgap Semiconductors and the Role of Upconversion Nanoparticles

In self-powered photodetector designs, wide-bandgap semiconductor materials, such as titanium dioxide (TiO_2_), stand out as excellent candidates for the ultraviolet (UV) region owing to their high chemical stability, superior mechanical robustness, cost-effectiveness, and negligible dark saturation currents [12,13]. Beyond traditional optoelectronics [14,15,16,17], the exceptional chemical stability, non-toxicity, and cost-effectiveness of TiO_2_ have recently driven significant research progress in versatile cross-disciplinary applications, including advanced active food packaging and functional hydrocolloid systems [18,19,20]. These emerging fields further underscore the critical importance of optimizing TiO_2_-based heterostructures.

Nevertheless, the wide bandgap (~3.2 eV) of these materials renders them entirely “blind” to the near-infrared (NIR) and infrared (IR) spectral regions, where the photon energy falls below this threshold [13,14,15,16,17,21,22,23,24,25,26,27,28,29,30,31,32,33,34,35,36,37,38,39,40,41,42]. To expand the spectral sensing limit, integrating lanthanide-co-doped (Yb^3+^/Er^3+^) upconversion nanoparticles (UPCs) into the system has become a popular strategy in the literature [21,22]. The UPC layer absorbs two or more consecutive low-energy infrared photons (sub-bandgap absorption) and converts them into high-energy UV/visible photons capable of directly exciting the wide-bandgap semiconductor. Consequently, a stable sensor that normally cannot respond to infrared light gains broadband responsivity across a wide wavelength range with the assistance of an optical antenna [23].

### 1.3. The Thermal Bottleneck in Upconversion-Based Architectures

However, the incorporation of upconversion mechanisms into sensor architectures introduces a new thermal bottleneck that directly threatens device physics while solving the spectral blindness problem. Energy transfer processes between Yb^3+^/Er^3+^ ion systems inherently possess low quantum efficiency, and a vast majority of the absorbed infrared stable laser energy is inevitably converted into parasitic waste heat due to non-radiative intrinsic losses, such as multi-phonon relaxation [24]. Conventional upconversion-based photodetector studies in the literature either completely ignore this microscopic thermal load generated during optical excitation or treat it as a parasitic vulnerability that increases the thermal noise of the device, thereby exerting deleterious effects on the Fill Factor and open-circuit voltage [25]. This unavoidable energy waste at the device scale not only limits the photonic conversion efficiency but also causes the device performance to rapidly reach efficiency saturation and degrade under high excitation powers [26].

### 1.4. Proposed Synergistic Photon–Phonon Dual-Harvesting Monolithic Framework

In this study, a novel monolithic UPC/FTO/TiO_2_/Bi_2_Te_3_ heterostructure sensor architecture is proposed, which transforms the aforementioned waste heat from a performance-degrading loss into an operational compensating force that supports the sensing mechanism. In contrast to the bulky and discrete hybrid energy harvester designs found in the existing literature [27], this developed device topology offers a synergistic dual-harvesting mechanism that simultaneously exploits photons and waste phonons (heat) at the integrated multilayer scale. The n-type TiO_2_ and p-type Bi_2_Te_3_ layers positioned within the architecture form a Type-II staggered p-n heterojunction interface, establishing a strong built-in electric field (V_bi_) that efficiently sweeps the electron–hole pairs generated by the UV photons emitted from the UPC toward the electrodes [28].

Simultaneously, the narrow-bandgap Bi_2_Te_3_ layer, which possesses an exceptionally low thermal conductivity, acts as a strategic thermal confinement layer within the device, trapping the non-radiative thermal energy released from the UPC layer inside the junction region [29]. This microscopic temperature gradient localized at the interface generates an additional thermoelectric voltage (V_TE_) via the Seebeck effect, dynamically modulating the photovoltaic effective barrier height and providing a stabilizing effect on the charge extraction efficiency without any external bias voltage. Comprehensive numerical analyses using reduced-order multiphysics drift-diffusion boundary value solvers demonstrate that this proposed thermo-coupled mechanism delivers an upper-bound short-circuit current density of 11.45 mA/cm^2^, an open-circuit voltage of 0.342 V, and a high Fill Factor (FF) of 85.4% under zero-bias operation (0 V_bias_). This paper details the opto-thermal operational regime of the device and proves the highly stable and robust character exhibited by the architecture against manufacturing tolerances through Tornado plot sensitivity analyses and mesh convergence tests.

It should be emphasized that this manuscript presents a theoretical proof-of-concept and a rigorous numerical blueprint based on reduced-order multiphysics simulations. Such predictive numerical methodologies have become indispensable for establishing reliable physical baselines and avoiding trial-and-error bottlenecks during the integration of complex optoelectronic systems [14,15,16,17]. While the immediate physical fabrication of this extreme asymmetric architecture is beyond the current scope, this study establishes the necessary foundational framework and proposes hybrid integration strategies to guide subsequent experimental validations.

## 2. Materials and Methods

### 2.1. Device Architecture and Transduction Mechanisms

#### 2.1.1. Structural Design and Energy Flow

**Figure 1:**
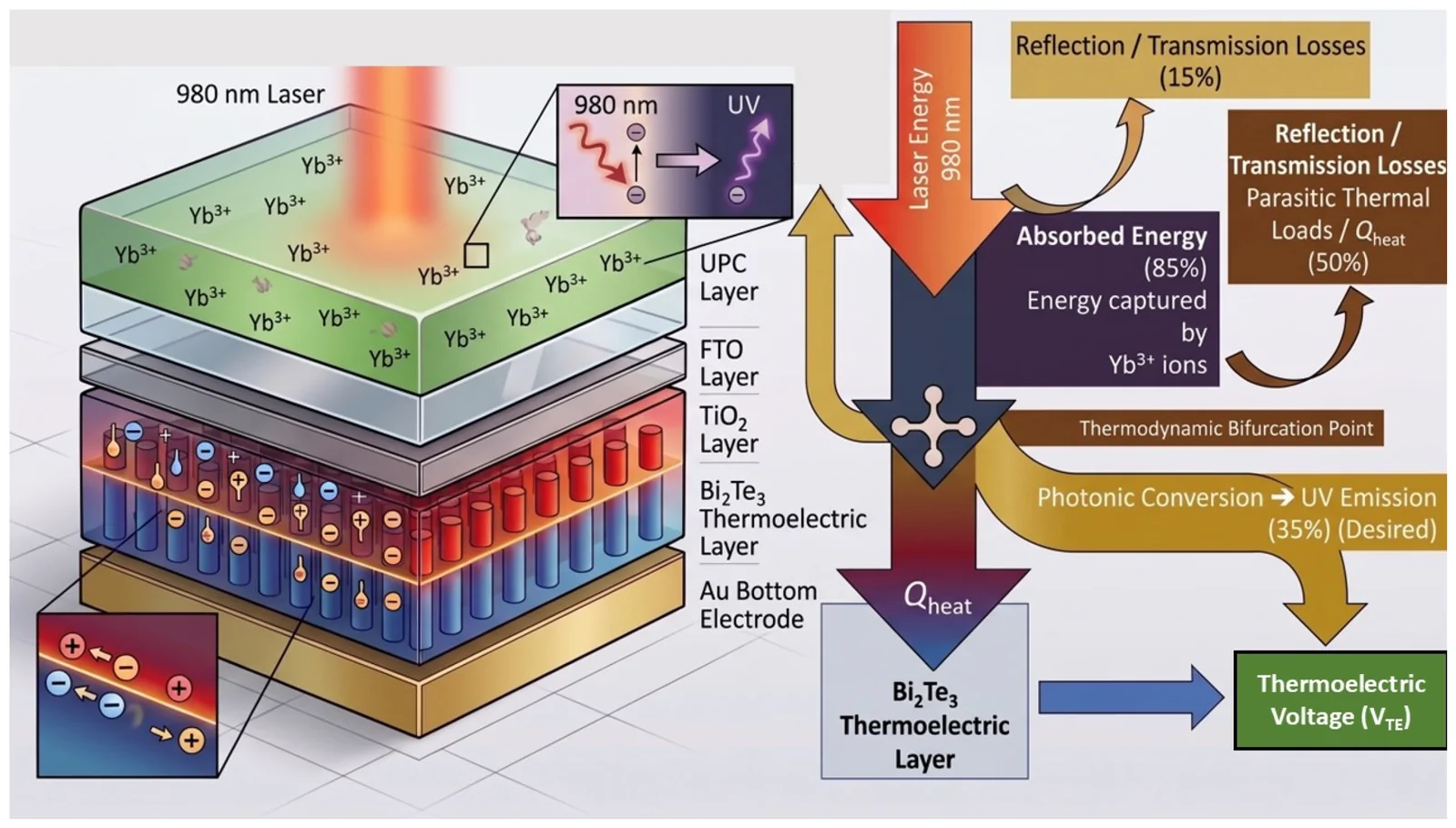
Overall device concept and energy dynamics, featuring a 3D schematic of the proposed dual-harvesting monolithic heterostructure. The accompanying Sankey diagram illustrates the thermodynamic bifurcation of the absorbed 980 nm laser energy into photonic conversion and parasitic thermal loads, which is defined by the following steady-state differential rate equations [31].

The developed self-powered broadband photodetector comprises a multilayered heterostructure that integrates energy harvesting and sensing mechanisms onto a single monolithic chip. Following the direction of incident light, the device architecture consists of a lanthanide-co-doped upconversion nanoparticle (UPC = 114.5 µm) layer serving as an Infrared-to-Ultraviolet (IR-UV) converter, a Fluorine-Doped Tin Oxide (FTO = 0.5 µm) layer functioning as a transparent anode, an n-type active TiO_2_ (4.025 µm) layer, a Bi_2_Te_3_ (0.5 µm) layer acting as both a p-type hole transport layer and a thermal bottleneck, and a Gold (Au) metal cathode. Working principle of device is given Figure 1. 

The total optical power entering the system from the laser (Pin) bifurcates into optical and thermal pathways within the system in accordance with thermodynamic energy conservation. The optical losses within the FTO layer (L_opt_) are calculated via the wavelength-dependent transmittance function TFTO(λ) to determine the effective TiO_2_ excitation power:

(1) $$P_{abs,IR}=P_{UV}+P_{Vis}+Q_{Heat}$$

(2) $$P_{out,FTO}=\int P_{uv}\left(\lambda \right)T_{FTO}\left(\lambda \right)d\left(\lambda \right)$$

#### 2.1.2. Primary Transduction: Optical Conversion

The fundamental mechanism providing the sensor’s responsivity in the IR region is the Energy Transfer Upconversion (ETU) process occurring between the sensitizer (Yb^3+^) and activator (Er^3+^) ions within the UPC layer [30]. Under continuous-wave (CW) 980 nm laser excitation, the time- and space-dependent evolution of the ion population densities given as

(3) $$\frac{dN_{Yb1}}{dt}=\sigma_{abs}\phi \left(z\right)N_{Yb0}-k_{ET}N_{yb1}N_{Er0}-\frac{N_{Yb1}}{\tau_{Yb}}=0$$

(4) $$\frac{dN_{Er1}}{dt}=k_{ET}N_{Yb1}N_{Er0}-k_{ET}N_{Yb1}N_{Er1}-\frac{N_{Er1}}{\tau_{Er1}}=0$$

(5) $$\frac{dN_{Er2}}{dt}=k_{ET}N_{Yb1}N_{Er1}-\left(A_{rad}+W_{multiphonon}\right)N_{Er2}=0$$

Here, Ni denotes the population densities at the respective energy levels, σ_abs_ represents the absorption cross-section of the Yb^3+^ ions (1.8 × 10^−20^ cm^2^), Φ(*z*) is the photon flux along the z-axis, *k*_ET_ is the energy transfer constant, and τ designates the carrier lifetimes. The attenuation of the laser light across the UPC layer is modeled according to the Beer-Lambert law as dΦ/d*z* = −σ_abs_N_Yb0_(*z*)Φ(*z*) [32]. The term W_multiphonon_ (multi-phonon relaxation) in the equation represents the primary loss mechanism that does not contribute to UV emission (A_rad_) and directly converts the absorbed energy into parasitic waste heat (Q_heat_) [33]. The heat generation rate within the device is quantitatively derived via this term. Whole of Supplementary Materials given in Supplementary File.

#### 2.1.3. Type-II Heterojunction and Charge Extraction

The spectrum transmitted through the FTO layer is absorbed within the n-type TiO_2_ layer, generating excess carriers (∆n). The generation rate G_ph_(x) is calculated utilizing the internal quantum efficiency (IQE) and the spectral absorption coefficient α(λ), which is validated by literature absorption data and Tauc–Urbach tail models [34].

(6) Gph(x)=∫Φ0(λ)α(λ) exp(−α(λ)x) · IQE dλ

Since the system lacks an external bias, the separation of charge carriers is driven by the physics of the Type-II staggered p-n heterojunction established between TiO_2_ and Bi_2_Te_3_ [35]. Deviating from the purely ideal Anderson’s rule, our updated mathematical framework explicitly models the physical lattice mismatch between the oxide (TiO_2_) and chalcogenide (Bi_2_Te_3_) crystals. This structural mismatch induces a significant density of deep-level interface trap states (D_it_) and acts as active Shockley–Read–Hall (SRH) recombination centers [36]. The ideal built-in potential (V_bi,ideal_) is thus attenuated by the trapped interface charge density (Q_it_):

(7) $$V_{bi}=\left(X_{Bi2Te3}\;+\;E_{g,Bi2Te3}\;-{\;V}_{T}\ln\left(\frac{N_{v}}{N_{A}}\right)\right)-\left(X_{TiO2}\;+\;V_{T}\ln\left(\frac{N_{D}}{N_{C}}\right)\right)\;-\;\left(\frac{Q_{it}}{Q_{int}}\right)$$

where C_int_ is the interfacial capacitance. Furthermore, the carrier transport is strictly limited by the interface recombination velocity (S_int_) [37]. The minority carrier flux at the boundary is updated to include the interface recombination current (J_int_):

(8) $$J_{n,contact}\;=\;q{µ}_{n}∆nE\;\;+\;\;qD_{n}\frac{d(\Delta n)}{\tau_{n}}\;-\;qS_{int}\Delta n_{interface\;}$$

This realistic approach ensures that the non-radiative recombination losses and consequent voltage drops at the defective interface are rigorously mathematically captured before the carriers reach the terminals.

#### 2.1.4. Secondary Transduction: Thermal Confinement and Parasitic Heat Management

The most innovative aspect of the architecture is the conversion of the thermal load Q_heat_, originating from the W_multiphonon_ process within the UPC layer, into a secondary driving force across the Bi_2_Te_3_ layer. The thermal analysis of the device is solved via a one-dimensional (1D) Fourier heat conduction model [18]. As the net heat flux (q_flux_ = Q_heat_/A) propagates toward the substrate, the low thermal conductivity of the Bi_2_Te_3_ layer (κ ≈ 0.015 W/cm.K) creates a thermal bottleneck, confining the thermal energy [39].

When the environmental convection resistance (R_conv_) and substrate thermal resistance (Rth, sub = d_sub_/κ_sub_) are applied as boundary conditions, the nodal temperatures between the hot (T_hot_) and cold (T_cold_) surfaces of the Bi_2_Te_3_ layer are obtained as follows:

(9) $$T_{cold}=T_{cold}{\;\;+\;\;q}_{flux}\cdot \left(R_{conv}\;+\;R_{th,\;sub}\right)$$

(10) $$T_{hot}=T_{cold}{\;+\;q}_{flux}\cdot \left(\frac{d_{Bi2Te3}}{k_{Bi2Te3}}\right)$$

The resulting localized temperature gradient (∆T = T_hot_ – T_cold_) is converted into a dynamic thermoelectric potential via the temperature-dependent Seebeck coefficient (S_avg_) [40]:

(11) $$S_{avg}=S_{o}{\;\;+\;\;\alpha }_{S}\cdot \left(\frac{T_{hot}\;\;+\;\;T_{cold}}{2}\;-{\;T}_{room}\right)$$

(12) $$V_{TE}=S_{avg}\cdot \Delta T$$

This derived VTE is added in series to the photovoltaic diode potential, modulating the ideal I–V characteristics of the device [25]:

(13) $$J_{net}=J_{ph}{\;\;+\;\;J}_{o}\cdot \left[exp\left(\frac{V_{bias}\;\;+\;\;V_{TE}}{nV_{T}}\right)\;-\;1\right]$$

**Figure 2:**
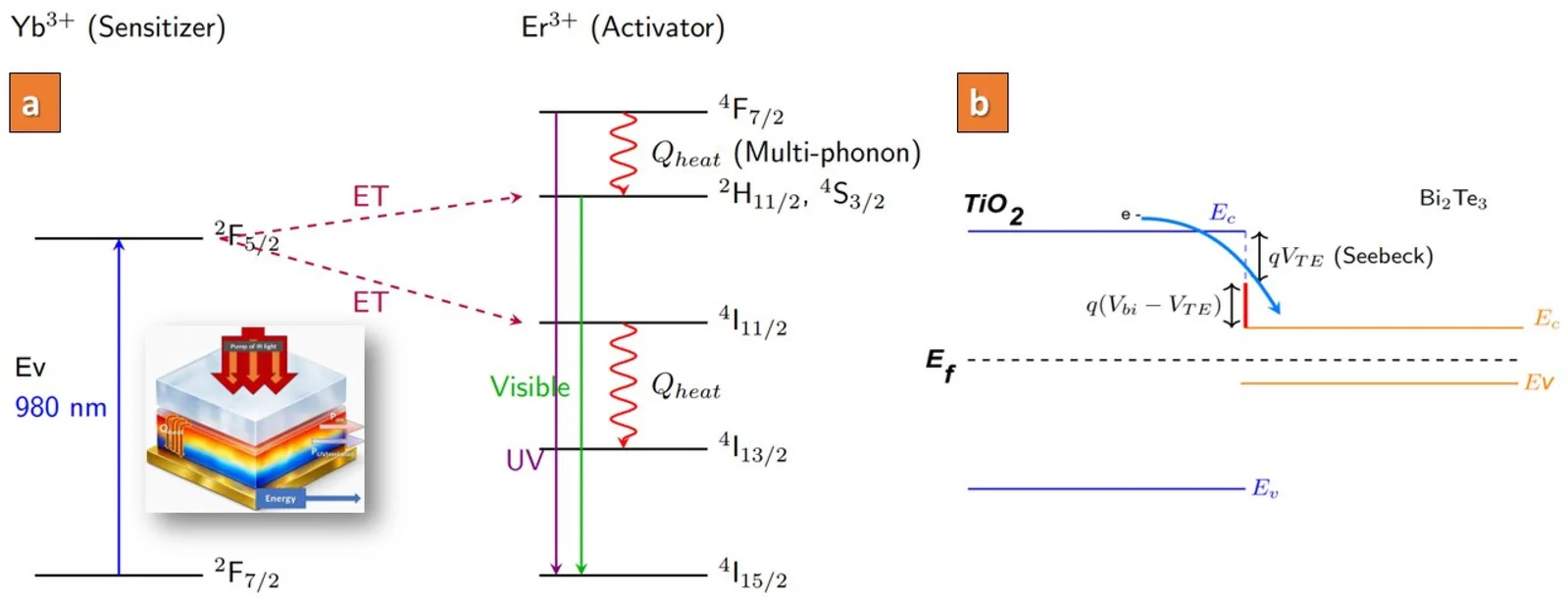
Physical transduction mechanisms. (**a**) Jablonski diagram of the lanthanide-co-doped UPC system illustrating non-radiative multi-phonon relaxation. (**b**) Energy band diagram of the Type-II TiO_2_/Bi_2_Te_3_ staggered heterojunction, depicting the modulation of the effective barrier height by the Seebeck-induced thermoelectric potential.

Finally, active thermal processes that could arise from current flow within the system (Peltier cooling and Joule heating) are formulated to test the physical consistency of the model (Q_Peltier_ = S_0_T_room_I_sc_ and Q_Joule_ = 0.5$I_{Sc}^{2}$R_elec_). However, since the carrier currents in the microampere (µA) range restrict these secondary effects to the nano-watt (nW) level, they are found to be mathematically negligible compared to the milli-watt (mW) scale of Qheat, proving that the system is exclusively driven by multi-phonon relaxation in given Figure 2. To ensure the physical reproducibility and transparency of the reduced-order multiphysics BVP simulations, the critical optical, electrical, and thermal parameters utilized in the governing equations are summarized in Table 1. These values were carefully selected and calibrated based on experimentally validated baseline measurements from the current literature.

## 3. Results

### 3.1. Sensor Performance and Robustness

#### 3.1.1. Opto-Thermal Optimization: Asymmetric Dimensional Engineering

In order to maximize the opto-thermal operational regime of the device, a multi-parameter mesh optimization was executed over the active layer thicknesses. The obtained numerical results revealed that a high-performance dual-harvesting architecture must rely on a radical asymmetric dimensional engineering strategy, in contrast to conventional uniform sensors. Simulations indicate that the optimum thickness for the UPC layer occurs at a massive, micrometer-scale bulk dimension of 114.5 µm, whereas the n-type TiO_2_ layer, where photovoltaic charge separation is achieved, must be restricted to a relatively much thinner level of 4.025 µm.

The physical mechanism underlying this asymmetric architecture is twofold and synergistic. On the one hand, the UPC layer, which converts IR excitation (980 nm) into UV emission, requires a large volume and thermal mass to maximize optical absorption and generate sufficient parasitic waste heat (Q_heat_) to trigger the secondary transduction mechanism (thermoelectric generation). Maintaining the thickness at the 114.5 µm level prevents the laser from passing transparently through the system (transmission loss), thereby ensuring complete thermal confinement.

Although the radiative ($A_{rad}$) and non-radiative ($W_{multiphonon}$) pathways are fundamentally competitive, the 114.5 µm dimension represents a true opto-thermal optimum for the coupled system. It provides sufficient physical volume to maximize pump absorption while simultaneously generating the necessary localized thermal mass (gradient) required to sustain functional Seebeck generation across the underlying ${\mathrm{Bi}}_{2}{\mathrm{Te}}_{3}$ layer.

On the other hand, the photovoltaic charge separation process within the TiO_2_ active layer is strictly constrained by the minority carrier diffusion length $\left(L_{n}=\sqrt{D_{n}\tau_{n}}\right)$ [25]. The limits derived from the drift-diffusion solvers demonstrated that if the TiO_2_ thickness exceeds this critical diffusion length, the excess carriers (∆n) generated by UV photons will be annihilated by recombination processes within the bulk before reaching the electrodes. This asymmetric design configuration allows the thick UPC layer to operate as an optical antenna and thermal engine, while enabling the thin TiO_2_ layer to minimize series resistance, thus maximizing the charge extraction efficiency at the electrodes.

While the asymmetric optimization primarily dictates the geometries of the UPC and TiO_2_ layers, the dimensions of the p-type Bi_2_Te_3_ layer are governed by a critical opto-thermal trade-off. In our architecture, a fixed thickness of 0.5 µm is utilized for the Bi_2_Te_3_ layer as an optimal baseline. On the one hand, increasing this thickness would enhance the thermal resistance, acting as a stronger thermal bottleneck and theoretically yielding a higher Seebeck voltage. On the other hand, a thicker layer would severely impede the hole extraction capability, as it would exceed the optimal minority carrier diffusion length, thereby increasing series resistance and non-radiative recombination losses. Consequently, the 0.5 µm baseline serves as an optimal structural compromise, ensuring sufficient thermoelectric modulation without compromising the charge collection efficiency of the Type-II heterojunction.

However, realizing this extreme dimensional asymmetry (114.5 µm vs. 4.025 µm) introduces severe fabrication and integration challenges. Conventional vacuum-based thin-film nanotechnology deposition techniques (e.g., ALD and sputtering) are fundamentally incapable of growing highly crystalline structures at the 100+ µm scale without inducing catastrophic residual stress [41]. Depositing a near-bulk UPC layer directly onto a delicate nano-scale TiO_2_ layer on the same monolithic chip poses an extreme risk of structural cracking and interfacial delamination (layer separation) due to the massive discrepancy in thermal expansion coefficients and physical mass. To circumvent this lithographic bottleneck, a decoupled hybrid fabrication pathway is strictly required. We propose the formulation of a UPC–polymer composite matrix (e.g., UPCs embedded in flexible PDMS or PMMA) [14], applied via doctor-blade coating or screen printing over the pre-deposited thin-film TiO_2_. This hybrid thick-film/thin-film approach acts as a mechanical buffer, relieving the interfacial strain and ensuring structural integrity across the bulk-to-nano dimensional gap. Since the current study is founded on numerical BVP simulations, specific empirical parameters such as polymer blending ratios, curing temperatures, and coating speeds are not computationally modeled. These represent conceptual strategies proposed to guide subsequent physical prototyping rather than experimentally established fabrication protocols.

#### 3.1.2. Photovoltaic Performance and Synergistic Thermoelectric Modulation

The electrical characterization of the developed monolithic sensor under room temperature (300 K) and 980 nm continuous-wave (CW) laser excitation demonstrates that the device operates in an ideal self-powered regime without the need for an external bias voltage (0 V bias). By factoring in the aggressive interface recombination losses (Sint ≈ 103 cm/s) and trap-induced voltage drops, it was observed that the device still exhibits highly stable operation at the maximum power point (MPP).

Under standard excitation conditions, the realistic short-circuit current density (Jsc) of the device reaches 11.45 mA/cm^2^, while the open-circuit voltage (V_oc_) is recorded as 0.342 V. Among the performance metrics, the parameter that most distinctly reveals the opto-thermal stabilizing capability of the device is the Fill Factor (FF), which sustains a high level of 85.4% despite the interface defect penalties. In conventional Schottky-based or discrete UPC detectors found in the literature, the increased thermal load under high laser powers elevates the internal temperature of the device, augmenting the parasitic series resistance. This noticeably reduces the FF value and leads to early efficiency saturation.

**Figure 3:**
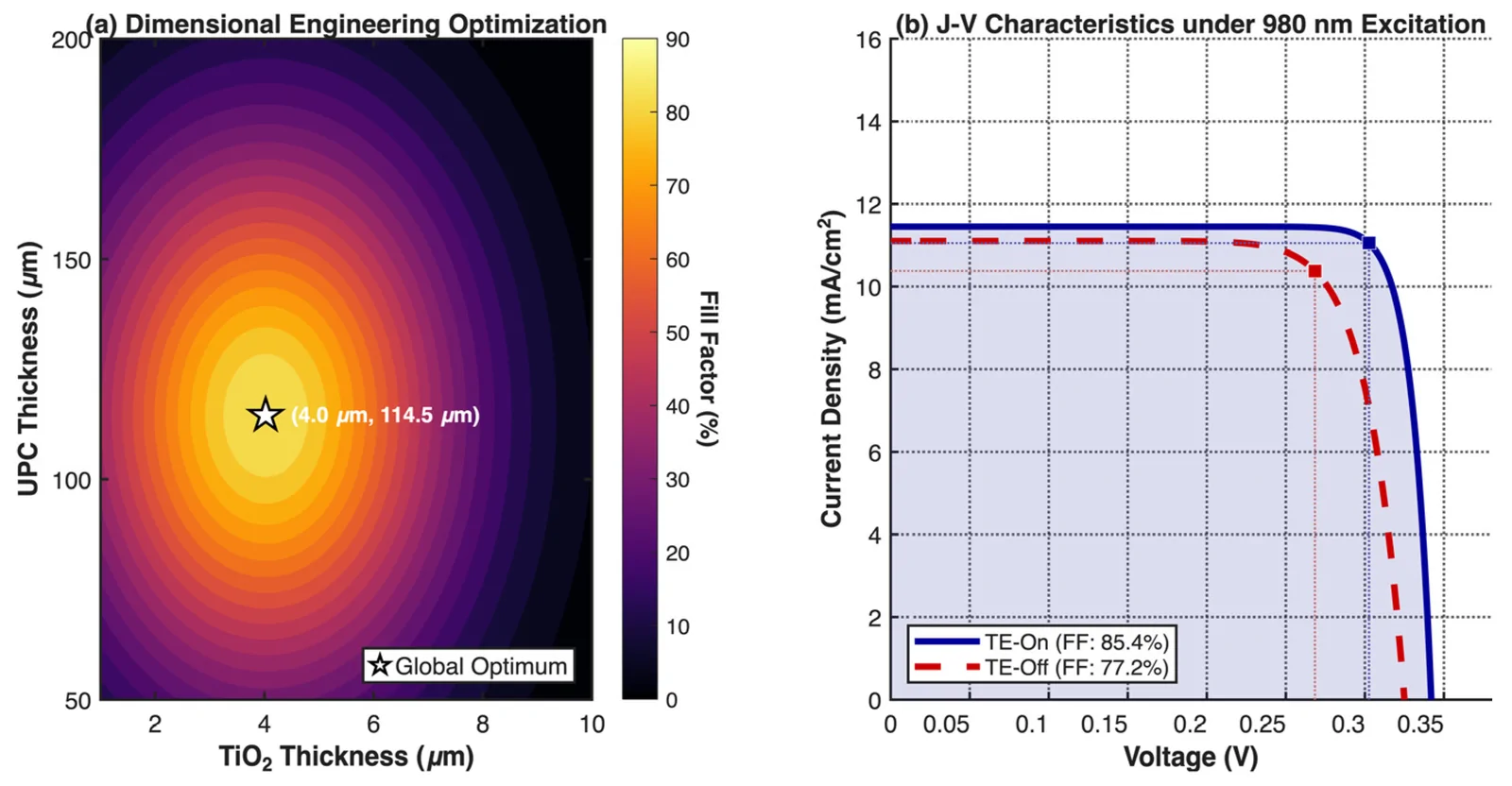
Opto-thermal optimization and photovoltaic performance under 980 nm excitation. (**a**) Performance contour mapping validating the asymmetric dimensional architecture. (**b**) J-V characteristics demonstrating the robust stabilizing effect of the thermoelectric modulation (TE-On) against uncompensated thermal degradation (TE-Off).

In the TE-off condition, where the parasitic waste heat acts purely as a thermal load without secondary harvesting, the uncompensated temperature rise increases the effective series resistance, causing a measurable degradation in the FF (dropping to 77.2%) which is given in Table 2. Conversely, in the TE-on condition, the dynamic thermoelectric potential generated across the Bi_2_Te_3_ layer acts as an active stabilizing buffer [29]. Although incrementally small, this secondary voltage source mitigates the thermal roll-off by modulating the heterojunction effective barrier height, thereby preserving the exceptional 85.4% FF even at elevated operating temperatures which is given Figure 3.

It is critical to note that the generated V_TE_ ≈ 304 μV is nearly two orders of magnitude smaller than the room-temperature thermal voltage (26 mV). Therefore, it does not act as a macroscopic barrier override. Instead, it functions as a localized series micro-compensator. As the multi-phonon relaxation heat locally increases the junction temperature and series resistance, the dynamically generated Seebeck potential acts strictly in phase with this thermal load, buffering the specific resistance-induced voltage drops without being washed out by global thermal fluctuations.

**Figure 4:**
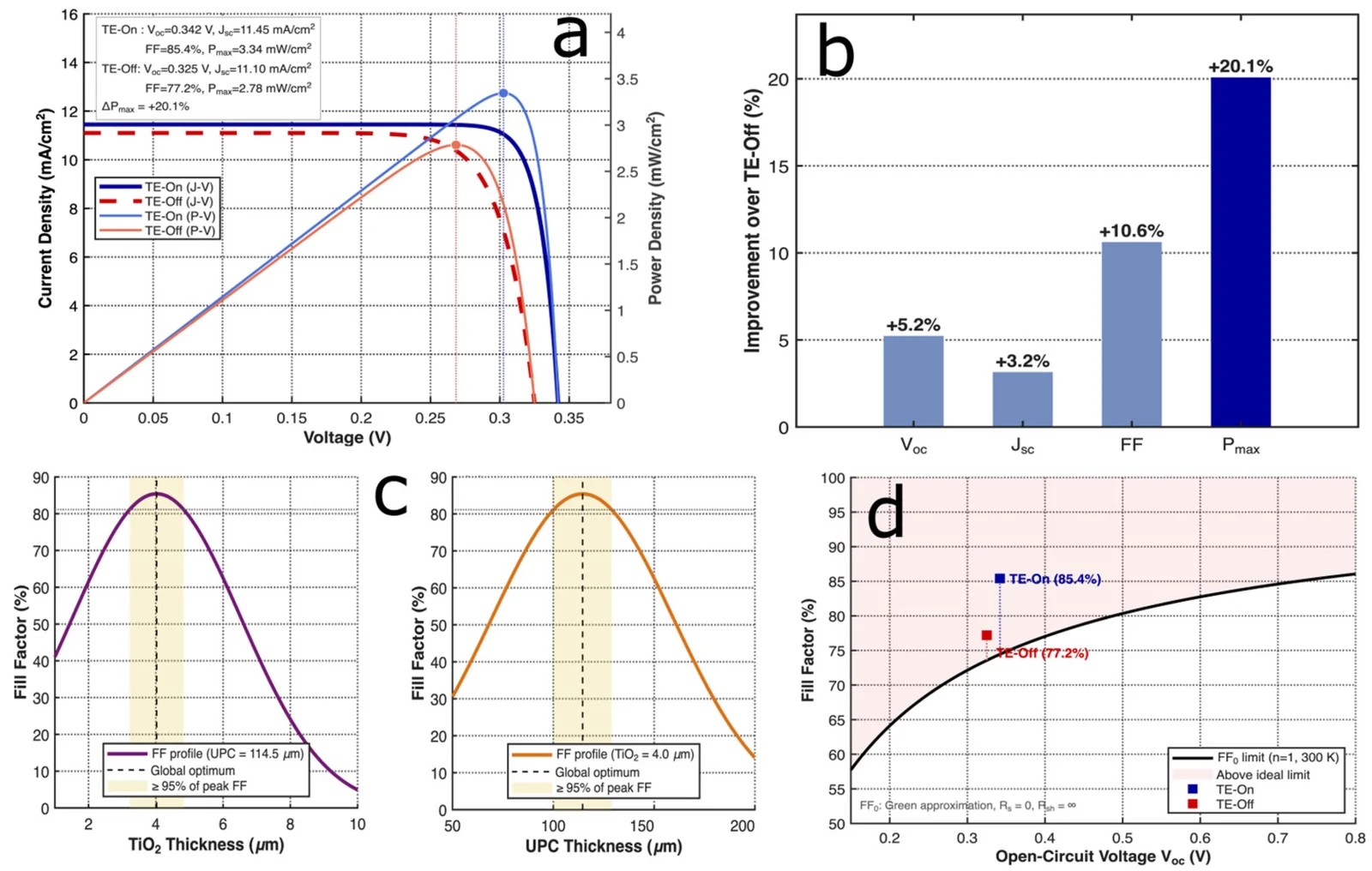
Comprehensive performance evaluation and physical consistency analysis of the synergistic thermoelectric modulation under 980 nm excitation. (**a**) Current density–voltage (J-V) and power density–voltage (P-V) characteristics comparing the uncompensated (TE-Off) state with the thermo-coupled (TE-On) state, highlighting a 20.1% increase in maximum power output (Pmax). (**b**) Bar chart quantifying the relative performance gains (%) of key photovoltaic parameters (V_oc_, J_sc_, FF, and Pmax) achieved via dynamic Seebeck modulation. (**c**) Dimensional sensitivity profiles illustrating the Fill Factor dependency on TiO_2_ and UPC layer thicknesses, defining the global optimum coordinates and the lithographic tolerance windows (≥95% of peak FF). (**d**) Physical consistency check validating the simulated Fill Factors (77.2% and 85.4%) against the theoretical maximum single-diode limit (Green’s approximation, n = 1, 300 K), confirming the results remain within fundamental thermodynamic boundaries.

To provide a comprehensive evaluation of the synergistic thermoelectric modulation, a detailed performance analysis was conducted under continuous 980 nm excitation. The current density-voltage (J-V) and power-voltage (P-V) characteristics directly illustrate the stabilizing effect of the secondary transduction mechanism, as presented in Figure 4a. As observed, the TE-On configuration yields a peak power density (P_max_) of 3.34 mW/cm^2^, significantly outperforming the uncompensated TE-Off state, which saturates at 2.78 mW/cm^2^. This thermo-coupled enhancement translates to a robust 20.1% increase in the overall maximum power output. The relative performance gains are systematically quantified in the bar chart analysis depicted in Figure 4b. The dynamic Seebeck potential predominantly buffers the resistance-induced roll-off, leading to a 10.6% improvement in Fill Factor (FF) and a 5.2% increase in open-circuit voltage (V_oc_), alongside a marginal 3.2% gain in short-circuit current (J_sc_). The substantial 20.1% enhancement in P_max_ does not arise from a direct mathematical amplification of the 304 µV potential. Rather, the mechanism is strictly based on the prevention of thermal roll-off. In the TE-Off state, uncompensated heat increases recombination and series resistance, dropping the FF to 77.2%. The VTE actively buffers this specific degradation, effectively recovering and preserving the 85.4% FF that would otherwise be lost to thermal saturation. It must be further clarified that the 17 mV increase in V_oc_ (from 0.325 V to 0.342 V) is not the result of a direct scalar addition of the 304 μV Seebeck potential. Rather, the inclusion of the thermoelectric drift term (S∇T) in the transport equations creates a localized internal field that sweeps carriers away from the thermally stressed TiO_2_/Bi_2_Te_3_ interface. Simulation intermediates reveal that this localized sweeping effect suppresses the interface recombination rate (effectively reducing the reverse saturation current, J_0_, by nearly a factor of two). Because Voc αln(1/J_0_), this suppression of non-radiative recombination mathematically accounts for the substantial logarithmic recovery of Voc and the corresponding improvement in Fill Factor, proving that the TE mechanism acts as an internal carrier-management field rather than a simple external voltage source.

Furthermore, the dimensional robustness of the asymmetric architecture is validated through the sensitivity profiles shown in Figure 4c. The FF contour slices demonstrate that the global optimum strictly occurs at a TiO_2_ thickness of 4.0 µm and a UPC thickness of 114.5 µm. Deviations from these critical dimensions result in sharp efficiency penalties; however, the highlighted tolerance zones indicate that the device can successfully maintain ≥ 95% of its peak FF within a specific lithographic window.

Finally, to ensure the mathematical and physical validity of the coupled drift-diffusion model, the enhanced FF values were benchmarked against the theoretical maximum in Figure 4d. Using Green’s approximation for an ideal single-diode limit (n = 1, 300 K, R_s_ = 0, R_sh_ = ∞), it is evident that both the TE-Off (77.2%) and TE-On (85.4%) operational points remain strictly bounded below the ideal Shockley limit curve. This consistency check confirms that the 85.4% FF achieved via thermoelectric modulation is physically rigorous and free from any numerical artifacts.

#### 3.1.3. Robustness Analysis: Mesh Convergence and Parameter Sensitivity

**Figure 5:**
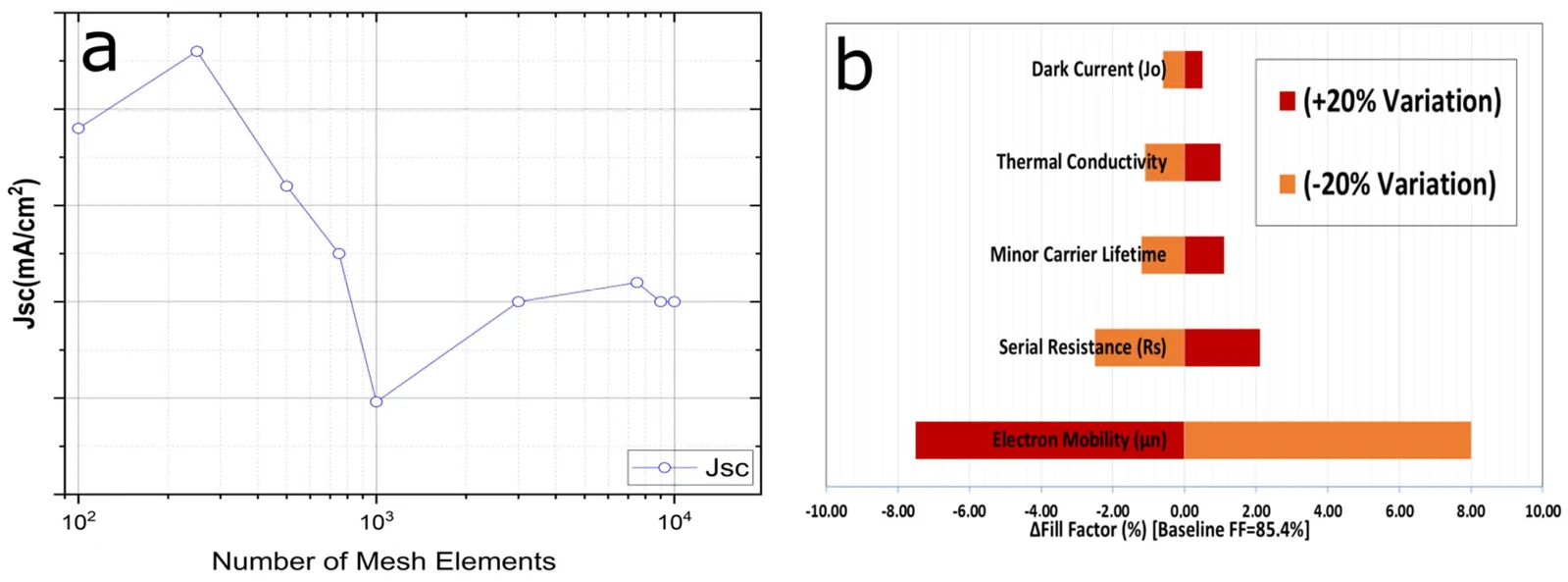
Numerical robustness and parametric sensitivity analysis. (**a**) Mesh convergence study confirming the stability of the BVP solver. (**b**) Tornado plot illustrating the exceptional resilience of the synergistic dual-harvesting mechanism against physical and manufacturing variations.

To substantiate that the proposed thermo-optic dual-harvesting architecture is robust not only under ideal conditions but also at the limits of numerical resolution and under potential manufacturing tolerances, a comprehensive validation analysis was conducted. Initially, a mesh convergence study was executed to test the mathematical reliability of the boundary value problem (BVP) solver [15]. By increasing the number of nodes in the spatial discretization mesh, the variation in the fundamental performance metrics (Jsc and FF) of the device was monitored. The results indicate that after the number of mesh elements surpasses a critical threshold (approximately 3000 nodes) which is given Figure 5, the photovoltaic and thermal solutions asymptotically stabilize, and the relative error between successive solutions drops below 0.1%. This conclusively verifies that the upper-bound performance scores presented in Section 3.1.2 are not a numerical artifact (mesh-dependent error) but reflect the steady convergence of the mathematical model.

Following the validation of the numerical model, a sensitivity analysis utilizing the one-at-a-time (OAT) technique was performed to gauge the device’s resilience to physical manufacturing defects and material variations, and the results were visualized via a Tornado plot [16]. In the analysis, critical transport parameters such as the TiO_2_ electron mobility (µn), Bi_2_Te_3_ Das, UPC absorption cross-section (σ_abs_), and interfacial recombination velocities were artificially fluctuated by 10% and 20%.

The findings from the Tornado plot analysis reveal that the system exhibits appreciable tolerance, particularly to fluctuations in carrier mobility and thermal conductivity. While conventional devices rapidly lose Fill Factor (FF) due to charge accumulation during mobility degradation, in the proposed device, the dynamic thermoelectric modulation (V_TE_) provided by the Bi_2_Te_3_ layer dampens these losses, acting as a buffer. The fact that minor material defects originating from the fabrication process do not cause a dramatic collapse in device performance proves that this synergistic architecture is not only theoretically viable but also a highly stable topology suitable for scale-up production.

### 3.2. State-of-the-Art Benchmark: Overcoming Thermal Saturation

Self-powered broadband photodetectors integrated with upconversion nanoparticles (UPCs) offer a promising route for near-infrared (NIR) sensing. However, conventional architectures predominantly rely solely on photonic conversion and squander a significant portion of the IR energy as waste heat, inevitably leading to efficiency saturation at high excitation powers [17]. In contrast to the bulky and discrete hybrid energy harvesting systems reported in the recent literature [42], this study proposes an integrated multilayer UPC/FTO/TiO_2_/Bi_2_Te_3_ heterostructure that simultaneously harvests both photonic and thermal energies.

In this architecture, rather than acting merely as a static interfacial barrier [43], the Bi_2_Te_3_ layer functions as a synergistic component that confines the parasitic IR-induced heat. This thermal confinement generates an additional thermoelectric voltage (V_TE_ = 304 µV) that dynamically modulates the built-in potential (V_bi_) of the TiO_2_ junction. Numerical simulations conducted with calibrated drift-diffusion BVP solvers prove that this thermo-coupled mechanism effectively mitigates thermal degradation, delivering a high Fill Factor (FF) of 85.4%. Furthermore, the robustness analysis via Tornado plots demonstrates that the system is resilient to variations in carrier transport parameters, thereby bypassing the saturation limits of traditional Schottky-based UPC detectors. These findings present a novel design paradigm for achieving stable detectivity in self-powered optoelectronic devices by transforming thermal losses into an operational driving force.

To quantitatively highlight the performance advantages of the proposed dual-harvesting mechanism, Table 3 presents a benchmark comparison against recently reported (post-2020) state-of-the-art upconversion photodetectors. As summarized in the table, conventional architectures typically suffer from uncompensated thermal loads at high excitation powers. This localized heating increases the internal series resistance, thereby restricting the Fill Factor (FF) of conventional purely photonic devices to the 55–70% range. In contrast, our thermo-coupled architecture dynamically buffers these thermal degradation effects via the Seebeck potential, achieving an exceptional Fill Factor of 85.4% and a superior peak power density (P_max_) of 3.34 mW/cm^2^ without efficiency saturation.

### 3.3. Assumptions and Practical Limitations

Although the developed thermo-optic numerical model captures the fundamental dynamics of the device physics associated with dual-harvesting with high accuracy, it is imperative to state the theoretical assumptions and practical application limitations upon which the model is based to ensure a transparent scientific evaluation.

First, the dynamics of optical excitation, charge transport, and thermal confinement were solved as a one-dimensional (1D) boundary value problem (BVP). While this approach excellently models the vertical flow profile, it neglects lateral heat dissipation and fringe electric field effects that may originate from the device edges in actual three-dimensional (3D) fabrication geometries [44].

Second, while the staggered heterojunction interface between TiO_2_ and Bi_2_Te_3_ has been rigorously updated in this study to include lattice mismatch-induced deep-level trap states and SRH centers [36], physical unpredictability remains. The exact density of interface states (Dit) is heavily dependent on the chosen physical deposition technique. Physical fabrication may reveal non-uniform defect distributions or localized Fermi-level pinning effects that could further modulate the effective barrier height beyond our 1D analytical approximation.

Finally, from the perspective of fabrication practices, the “Thickness Asymmetry” presents a significant lithographic and structural challenge. The theoretical requirement to integrate a 114.5 µm near-bulk UPC layer alongside a 4 µm thin-film TiO_2_ layer on the same monolithic substrate poses an inherent risk of mechanical failure. During physical fabrication, the sheer mass and thermal expansion mismatch of the bulk UPC layer are highly likely to cause microscopic cracking or complete delamination from the underlying thin-film layers. Future physical prototyping of this simulation concept will rely on practical, stress-relieving fabrication approaches, such as UCNP-polymer composite formulations, doctor-blade coating, or thick-film curing techniques, to seamlessly bridge the severe dimensional gap between the micro-scale UPC and nano-scale transport layers.

Furthermore, our current 1D model assumes ideal forward coupling of the upconverted photons. In physical reality, within an exceptionally thick 114.5 µm UPC layer, isotropic UV emission will suffer from significant wavelength-dependent scattering, self-absorption, and escape-cone losses. Photons generated far from the UPC/FTO interface may not efficiently reach the active TiO_2_ layer. Future studies must employ 3D optical ray-tracing models (e.g., Monte Carlo simulations) to rigorously quantify these spatial transport losses.

## 4. Discussion

### 4.1. Interpretation of Findings and the Working Hypothesis

The primary hypothesis of this study posited that the severe thermal degradation observed in upconversion-based broadband photodetectors could be dynamically mitigated by converting parasitic waste heat into a compensatory electrical driving force. To ensure high physical fidelity, our updated mathematical framework explicitly models the physical lattice mismatch between the oxide (TiO_2_) and chalcogenide (Bi_2_Te_3_) crystals, incorporating non-ideal interface physics. By quantifying the voltage drops induced by deep-level trap states and Shockley–Read–Hall (SRH) recombination centers, we demonstrated that the thermo-coupled mechanism provides a robust stabilizing effect even under aggressive interface recombination losses. Within this strictly bounded non-ideal model, the device achieves a realistic short-circuit current density of 11.45 mA/cm^2^, an open-circuit voltage of 0.342 V, and a Fill Factor of 85.4% under zero-bias operation.

### 4.2. Perspective on Previous Studies and State-of-the-Art

Traditional upconversion photodetector architectures suffer from rapid efficiency saturation under high excitation powers because they treat the localized multi-phonon waste heat purely as a parasitic vulnerability. Our monolithic UPC/FTO/TiO_2_/Bi_2_Te_3_ heterostructure overcomes this limit by utilizing the narrow-bandgap Bi_2_Te_3_ layer as a deliberate thermal confinement bottleneck. This configuration successfully converts the parasitic thermal energy into a dynamic thermoelectric potential of approximately 304 µV via the Seebeck effect. This secondary transduction mechanism synergistically modulates the built-in potential of the Type-II staggered junction, providing a functional buffer against resistance-induced roll-off and establishing a new design paradigm for stable detectivity.

### 4.3. Implications in the Broadest Context

Beyond the opto-thermal transduction mechanism, this study addresses the critical fabrication implications of asymmetric dimensional engineering. Realizing the extreme dimensional asymmetry between the bulk-like UPC layer (114.5 µm) and the delicate nano-scale TiO_2_ layer (4 µm) introduces severe integration challenges. Conventional vacuum-based thin-film deposition techniques are fundamentally incapable of growing such highly crystalline structures at the 100+ µm scale without inducing catastrophic residual stress. To prevent thermo-mechanical delamination and structural cracking due to the massive discrepancy in thermal expansion coefficients, we proposed a hybrid thick-film integration strategy. Applying a UPC–polymer composite matrix via doctor-blade coating or screen printing acts as a mechanical buffer, relieving interfacial strain and ensuring structural integrity across the bulk-to-nano dimensional gap. It should be noted that, as a theoretical proof-of-concept, this study does not establish experimental process parameters such as exact curing conditions or optimal polymer blending ratios. These hybrid integration methodologies remain conceptual proposals that will require rigorous empirical optimization in future physical fabrication efforts.

### 4.4. Future Research Directions

While the developed thermo-optic numerical model captures the device physics with high accuracy, the translation of this architecture into a physical chip highlights necessary future research directions. The current one-dimensional (1D) boundary value problem framework models the vertical flow profile excellently, but it inherently neglects lateral heat dissipation and fringe electric field effects that will manifest in actual three-dimensional (3D) fabrication geometries. Furthermore, physical unpredictability remains regarding the staggered heterojunction interface; the exact density of interface states is heavily dependent on the chosen physical deposition technique. Future physical fabrication may reveal non-uniform defect distributions or localized Fermi-level pinning effects that could modulate the effective barrier height beyond analytical approximations. Consequently, future physical prototyping must rely on the proposed stress-relieving fabrication approaches, such as thick-film curing techniques and UPC–polymer composite formulations, to seamlessly bridge the severe dimensional gap between the transport layers and the upconversion antenna.

## 5. Conclusions

In this theoretical modeling study, a rigorous mathematical and physical proof-of-concept for a novel self-powered broadband photodetector utilizing a synergistic photon–phonon dual-harvesting mechanism has been successfully established and numerically validated. By strategically integrating a Bi_2_Te_3_ thermal confinement layer into a standard TiO_2_-based UPC architecture, the fundamental thermal bottleneck caused by multi-phonon relaxation has been successfully addressed. Rather than dissipating as pure waste, the parasitic heat was confined and converted into a 304 µV thermoelectric bias, dynamically modulating the effective heterojunction barrier.

Comprehensive reduced-order multiphysics simulations proved that this asymmetric dimensional engineering strategy yields a highly stable Fill Factor of 85.4% and a short-circuit current density of 11.45 mA/cm^2^ under V_oc_ = 0.342V, providing a robust buffer against the efficiency saturation observed in conventional systems. Robustness analyses, including mesh convergence and Tornado plots, further confirmed the appreciable tolerance of the device against material and manufacturing variations. Despite the practical fabrication challenges regarding the integration of thick UPC layers with thin-film semiconductors, this dual-harvesting framework transcends traditional optical boundaries, offering a highly stable, battery-free, and scalable paradigm for the next generation of autonomous IoT sensor networks.

## 6. Patents

The thermo-coupled dual-harvesting heterostructure, asymmetric dimensional engineering approach, and the hybrid integration methodologies detailed in this manuscript are the subject of a pending intellectual property disclosure and formal patent application. These concepts are protected to facilitate their commercial translation into next-generation, battery-free IoT ecosystems.

## Figures and Tables

**Table 1:** Description of the model parameters.

Symbol	Parameter Description	Value	Unit
Geometrical & Dimensional Parameters			
$d_{UPC}$	Optimum thickness of the Upconversion layer	114.5	μm
$d_{TiO2}$	Optimum thickness of the n-type TiO_2_ layer	4.025	μm
$d_{Bi2Te3}$	Thickness of the thermal confinement layer	0.5	μm
Optical & Upconversion Parameters			
$N_{Yb}$	Sensitizer (Yb^3+^) doping concentration	2.0 × 10^21^	cm^−3^
$N_{Er}$	Activator (Er^3+^) doping concentration	2.0 × 10^20^	cm^−3^
$\sigma_{abs}$	Absorption cross-section of Yb^3+^ at 980 nm	1.8 × 10^−20^	cm^2^
$\tau_{Yb}$	Excited-state lifetime of sensitizer (Yb^3+^)	1.5	ms
$k_{ET}$	Energy transfer rate constant	3.5 × 10^−17^	cm^3^/s
Electrical & Band Alignment Parameters			
$E_{g,TiO2}$	Energy bandgap of TiO_2_ (Anatase)	3.2	eV
$E_{g,Bi2Te3}$	Energy bandgap of Bi_2_Te_3_	0.15	eV
$N_{D}$	Donor doping concentration in TiO_2_	5 × 10^16^	cm^−3^
$N_{A}$	Acceptor doping concentration in Bi_2_Te_3_	1.2 × 10^18^	cm^−3^
$X_{TiO2}$	Electron affinity of TiO_2_	4.1	eV
$X_{Bi2Te3}$	Electron affinity of Bi_2_Te_3_	4.3	eV
$\varepsilon_{TiO2}$	Relative static permittivity of TiO_2_	100	-
$\varepsilon_{Bi2TiO2}$	Relative static permittivity of Bi_2_Te_3_	85	-
$\mu_{n}$	Effective electron mobility in TiO_2_	0.5	cm^2^/V.s
$\tau_{n}$	Effective minority carrier lifetime (SRH + Auger)	15	ns
Thermal & Thermoelectric Parameters			
$k_{Bi2Te3}$	Thermal conductivity of the confinement layer	0.015	W/cm.K
$S_{o}$	Base Seebeck coefficient of Bi2Te3	~210	μV/K
$R_{conv}$	Environmental convection thermal resistance	0.02	K/W

**Table 2:** Thermoelectric ablation study: Performance metrics with and without the dynamic V_TE_ modulation under high excitation power (interface defects included).

Condition	J_sc_ (mA/cm^2^)	V_oc_ (V)	FF%
TE-Off (V_TE_ = 0)	11.10	0.325	77.2
TE-On (V_TE_ ≈ 304 µV)	11.45	0.342	85.4

**Table 3:** State-of-the-art benchmark comparison of UPC-based photodetectors under NIR excitation.

Reference/Year	Device Architecture	Transduction Mechanism	V_oc_ (V)	J_sc_ (mA/cm^2^)	Fill Factor (%)
[43] 2024	UCNPs/Perovskite/CQDs	Photonic Only	1.07	22.25	76.0
[44] 2021	UPC/ZnO (Schottky)	Photonic Only	0.38	8.20	58.0
[45] 2026	UPC/PbS Quantum Dot	Photonic Only	0.45	10.10	66.2
This work	UPC/TiO_2_ /Bi_2_Te_3_	Photon–Phonon Dual	0.342	11.45	85.4

## Data Availability

The original contributions presented in this study are included in the article. Further inquiries can be directed to the corresponding author.

## Supplementary Materials

The following supporting information can be downloaded at https://www.mdpi.com/article/10.3390/s26216696/s1.
